# Assessment of Awareness and Knowledge Regarding Dental Implants Among Adult Partially Edentulous Patients Attending a Dental Teaching Institution

**DOI:** 10.7759/cureus.117627

**Published:** 2026-10-08

**Authors:** Niharika Bishnoi, Vineet Galhotra, Taranjit Singh Walia, ARV Gayathrinath, Fatinderjeet Singh, Ashish Kakkar

**Affiliations:** 1 Oral and Maxillofacial Surgery, Private Practice, Hanumangarh, IND; 2 Dentistry, Dayanand Medical College & Hospital, Ludhiana, IND; 3 Prosthodontics, Teerthanker Mahaveer University, Moradabad, IND; 4 Prosthodontics, Private Practice, Telangana, IND; 5 Endodontics, Teerthanker Mahaveer University, Moradabad, IND; 6 Dentistry, Ex-Servicemen Contributory Health Scheme (ECHS), Fatehabad, IND

**Keywords:** awareness, dental implants, knowledge, missing teeth, patient attitude

## Abstract

Background

Dental implants are a predictable and effective treatment option for replacing missing teeth, offering superior functional and esthetic outcomes. However, successful acceptance of implant therapy largely depends on patients' knowledge and awareness. This study assessed the knowledge, awareness, and attitudes regarding dental implants among dental patients.

Materials and methods

A hospital-based cross-sectional questionnaire survey was conducted among 5,000 partially edentulous dental patients aged ≥ 18 years attending the outpatient department of a dental teaching institution. Participants were recruited using consecutive sampling. A pretested structured questionnaire comprising 18 closed-ended questions assessed sociodemographic characteristics and knowledge, awareness, and attitudes regarding dental implants. Data were analyzed using IBM Statistical Package for the Social Sciences (SPSS) version 21.0 (IBM Corp., Armonk, NY) using descriptive statistics, and binary logistic regression analysis was done to assess factors associated with knowledge of dental implants.

Results

Of the 5,000 eligible participants, 4,700 completed the questionnaire (response rate: 94%). A history of tooth extraction was reported by 3,358 (71.4%) participants, with dental caries being the most common cause (1,805; 53.8%). Among those with missing teeth, 3,102 (92.4%) expressed a desire for tooth replacement, and 1,309 (42.2%) preferred dental implants. Awareness regarding dental implants was reported by 1,909 participants, while 1,193 were unaware of this treatment option. Most respondents perceived implant therapy as expensive, with 1,341 (28.5%) estimating the treatment cost to be ₹10,000-₹20,000 and 1,100 (23.4%) believing it exceeded ₹20,000 or varied by implant system. Regarding maintenance, 2,612 participants believed implants require different oral hygiene care than natural teeth, whereas 1,298 considered the maintenance requirements similar.

Conclusion

The study demonstrated moderate awareness and a generally positive attitude toward dental implants among dental patients. Dentists were identified as the primary source of information, emphasizing their pivotal role in patient education. Improving public awareness, strengthening patient counseling, and reducing treatment costs through supportive health policies may enhance the acceptance of implant therapy.

## Introduction

Tooth loss is a prevalent oral health condition resulting from multiple etiological factors, including dental caries, periodontal diseases, maxillofacial trauma, endodontic treatment failure, and occasionally iatrogenic causes [1]. Beyond the functional deficit, edentulism constitutes a major adverse life event with substantial psychological and social implications, often requiring significant emotional adaptation and lifestyle modification. The World Health Organization recognizes complete edentulism as a physical impairment because of the irreversible loss of an essential component of the stomatognathic system [2].

Edentulous individuals commonly experience compromised masticatory efficiency, impaired speech articulation, diminished facial support, reduced dentofacial aesthetics, and psychosocial discomfort that may adversely affect overall quality of life [3,4]. Conventional prosthodontic rehabilitation has primarily relied on removable partial dentures, fixed dental prostheses, and complete dentures for patients with partial or complete edentulism [5].

In contemporary dental practice, osseointegrated dental implants have emerged as a predictable and effective therapeutic modality for the replacement of missing teeth in both partially and completely edentulous patients [5]. Inadequate patient education and ineffective clinician-patient communication regarding available treatment options may contribute to misconceptions, reduced self-confidence, and poor treatment acceptance [6]. Conversely, comprehensive patient counseling enhances awareness and facilitates informed decision-making. The selection of an appropriate tooth replacement modality is influenced by multiple clinical and patient-related determinants, including aesthetic requirements, the location and extent of tooth loss, alveolar ridge morphology, quantity and quality of supporting bone, age, sex, socioeconomic status, financial affordability, systemic considerations, and individual treatment preferences [7]. Against this background, the present study was undertaken to evaluate the knowledge, awareness, and attitudes regarding dental implants as a treatment option for the replacement of missing teeth among dental patients.

## Materials and methods

This institution-based cross-sectional questionnaire survey was conducted among 5,000 adult dental patients attending the general outpatient department (OPD) during the study period of six months. The study was conducted at a dental teaching institution located in Moradabad, Uttar Pradesh, India. Participants were recruited from the general dental OPD and not exclusively from the Department of Prosthodontics. The study protocol was reviewed and approved by the Institutional Review Board/Ethics Committee of the institution. Prior to enrolment, participants received a detailed participant information sheet in their preferred language (English/Hindi), and written informed consent was obtained from all participants.

A non-probability consecutive sampling technique was employed. All eligible adult dental patients attending the OPD during the study period were invited to participate until the predetermined sample size of 5,000 participants was achieved.

The study population comprised adult dental patients with partial edentulism, defined as the presence of one or more missing natural teeth with at least one natural tooth remaining in the dentition. Thus, all 5,000 participants included in the final study population fulfilled the criterion of partial edentulism. A total of 5,000 participants were initially assessed/enrolled, of whom 4,700 participants met the eligibility criteria and had complete data available for analysis. Therefore, all analyses and results presented in the manuscript are based on the final analytical sample of 4,700 participants. Participants with no previous history of tooth extraction were not eligible for inclusion if they did not have partial edentulism or a missing tooth requiring replacement. The questionnaire also recorded the history of previous tooth extraction and participants' preferred treatment options for replacement of missing teeth. The questionnaire was administered to eligible dental patients irrespective of whether they had been specifically referred to the prosthodontics department; recruitment was performed through the general dental OPD.

The study did not require participants to have a previously established clinical indication for dental implant therapy specifically. Rather, the study assessed their awareness, knowledge, attitudes, treatment preferences, and willingness regarding dental implants as a potential treatment option for replacing missing teeth. Clinical suitability for implant placement was not independently determined as an inclusion criterion, and the study findings therefore represent awareness and perceptions regarding dental implants rather than the prevalence of patients clinically eligible for implant therapy.

The sample size was calculated using the single population proportion formula, considering a 95% confidence level, 5% absolute precision, and an expected awareness proportion based on previous literature, with additional recruitment to compensate for incomplete responses and enhance the precision of the estimates.

The sample size was calculated using the single-population proportion formula:

$n=\frac{Z^2p(1-p)}{d^2}$

where n is the required sample size, Z is the standard normal deviate at a 95% confidence level (1.96), p is the anticipated prevalence, and d is the desired absolute precision.

For the calculation, an anticipated prevalence of 50% was assumed, representing a conservative estimate in the absence of a precise prior estimate. An absolute precision of approximately 1.51% was used with a 95% confidence level. The resulting minimum sample size was approximately 4,225 participants. Allowing for approximately 10% non-response or incomplete data, the required sample size was approximately 4,700 participants. Accordingly, 5,000 participants were initially assessed, of whom 4,700 participants with eligible and sufficiently complete data were included in the final analytical sample.

Participants aged 18 years or older with partial edentulism who were willing to participate were included in the study. Individuals with complete edentulism, physical disabilities that limited questionnaire completion, and those associated with the dental profession, including dental surgeons, dental assistants, dental hygienists, dental technicians, and dental students, were excluded to minimize professional knowledge bias.

A structured, pretested questionnaire was modified to suit the characteristics of the study population. A structured, pretested questionnaire was modified to suit the characteristics of the study population. The questionnaire was modified from Al-Dwairi et al. and comprised 18 closed-ended items organized into two sections: (i) socio-demographic characteristics, including age, gender, and educational status, and (ii) knowledge, awareness, and attitudes regarding dental implants as a treatment modality for replacing missing teeth [6]. The questionnaire also collected information regarding previous tooth extraction history, preferred treatment options, awareness of dental implants, sources of information, and willingness to undergo implant therapy.

The questionnaire was made available in both English and Hindi to facilitate comprehension among participants. The English version was translated into Hindi by a bilingual language expert familiar with medical terminology. The Hindi version was subsequently back-translated into English by an independent bilingual translator who was not involved in the initial translation. 

Potential confounding variables, including age, sex, educational status, and socioeconomic background, were recorded and considered during data interpretation because these factors may influence the level of awareness and treatment preferences regarding dental implants.

Completed questionnaires were checked for completeness and entered into Microsoft Excel (Microsoft Corporation, Redmond, WA) before being exported to IBM Statistical Package for the Social Sciences (SPSS) version 21.0 (IBM Corp., Armonk, NY) for statistical analysis. Descriptive statistics were used to summarize the data as frequencies and percentages. Binary logistic regression analysis was performed to assess factors associated with knowledge of dental implants.

## Results

Out of 5000 patients, 4700 participated positively in this study. As a result, the response rate was determined to be 94%. The individuals who did not complete the questionnaire were excluded, and any incomplete data were disregarded in the survey. In terms of gender distribution, the sample comprised 4700 individuals, with 2458 identified as men and 2242 as women. Men were marginally more represented than women. A significant portion of the overall sample consisted of younger individuals, specifically those under the age of 30 years, totalling 2250 out of 4700. Additionally, 1754 participants fell within the age range of 30-50 years. The educational background of the patients was also evaluated, revealing that 1601 out of 4700 held a bachelor's degree, while 1344 out of 4700 had attained secondary education or lower, as shown in Table 1.

**Table 1:** Distribution of the study population according to sociodemographic variables (n = 4700)

Variables	Category	n (%)
Age (years)	<30	2,250 (47.9%)
	30–50	1,754 (37.3%)
	>50	696 (14.8%)
Gender	Male	2,458 (52.3%)
	Female	2,242 (47.7%)
Educational level	High school or below	1,344 (28.6%)
	Diploma holder	1,048 (22.3%)
	Bachelor's degree	1,601 (34.1%)
	Master's degree	584 (12.4%)
	PhD	123 (2.6%)

As shown in Table 2, 3358 out of 4700 patients reported having undergone tooth extraction. Upon analyzing the primary reason for these extractions, 1805 individuals identified tooth decay or dental caries as the predominant cause. A significant majority of the patients (3102 out of 3358) expressed a desire for the replacement of their missing teeth. When inquired about their preferred method of replacement, 1309 participants showed a favorable inclination toward dental implants as a viable option. Among the dental patients surveyed, 1909 acknowledged having knowledge of dental implants, whereas 1193 respondents were unaware of them. 

**Table 2:** Survey questions on patients' knowledge and attitude regarding dental implants

Questions	Response	n (%)
Do you have any history of tooth extraction?	Yes	3,358 (71.4%)
	No	836 (17.8%)
	Can't say	506 (10.8%)
If yes, what was the reason for extraction/loss of tooth?	Tooth decay	1,805 (53.8%)
	Gum disease	985 (29.3%)
	Trauma	365 (10.9%)
	Other	203 (6.0%)
When did you get the tooth extracted?	0–5 years	1,214 (36.2%)
	5–10 years	1,111 (33.1%)
	10–15 years	703 (20.9%)
	>15 years	330 (9.8%)
Do you want to replace the missing tooth?	Yes	3,102 (92.4%)
	No	256 (7.6%)
What replacement do you need in the extracted tooth region?	Removable partial denture (RPD)	502 (16.2%)
	Fixed partial denture (FPD)	607 (19.6%)
	Dental implants	1,309 (42.2%)
	Don't know	684 (22.1%)
Have you ever heard anything related to dental implants?	Yes	1,909 (61.5%)
	No	1,193 (38.5%)
What was your first source of information about dental implants?	Dentists	1,807 (58.3%)
	Relatives and friends	963 (31.0%)
	Family doctor	214 (6.9%)
	Internet	118 (3.8%)
What are the reasons for not choosing dental implants as a treatment option?	Very expensive treatment	564 (31.4%)
	Takes a long time	423 (23.5%)
	Fear/anxiety	415 (23.1%)
	Needs surgery	266 (14.8%)
	Lack of information about implants	125 (7.0%)

Most participants perceived dental implant treatment as one of the most expensive ​​​​​dental procedures, with 1341/4700 patients (32%) affirmatively stating that the treatment cost ranges from ₹10,000 to ₹20,000. Conversely, 1100 patients (19%) believed ​​​​​​​that the expense of dental implant treatment could exceed ₹20,000 or differ based on the brand.​​​​​​​ Regarding implant care and hygiene compared to natural teeth, 2612 subjects thought that implants require more or less care and maintenance of oral hygiene than natural teeth. Moreover, 1298 subjects believed that implants might need similar oral hygiene care as compared to natural teeth, as shown in Table 3.

**Table 3:** Survey questions on patients' awareness regarding dental implants

Questions	Response	n (%)
According to you, what do you estimate as the functional life of implants?	<10 years	856 (18.2%)
	10–20 years	1,959 (41.7%)
	21–25 years	965 (20.5%)
	>25 years	482 (10.3%)
	No idea	438 (9.3%)
Up to what amount are you prepared to pay as an additional payment for an implant?	₹5,000	1,001 (21.3%)
	₹10,000–₹20,000	1,341 (28.5%)
	>₹20,000	1,100 (23.4%)
	Varies from brand to brand (commercial availability of implant)	1,258 (26.8%)
What do you anticipate oral hygiene for the care of implants as compared with natural teeth?	More than natural teeth	1,589 (33.8%)
	Less than natural teeth	1,023 (21.8%)
	Similar to natural teeth	1,298 (27.6%)
	Can't say	790 (16.8%)
What are you expecting after getting treatment with an implant as an option for replacing missing teeth?	Long-lasting	2,689 (57.2%)
	Better aesthetics	1,487 (31.6%)
	Less maintenance	524 (11.1%)
Would you be interested in knowing more about dental implants?	Yes	3,954 (84.1%)
	No	746 (15.9%)

The awareness analysis included 3,102 participants who provided a valid response to the specific awareness question. Of these, 1,909 (61.5%) reported awareness, while 1,193 (38.5%) reported no awareness. The remaining 1,598 participants had no valid response and were therefore excluded from this question-specific analysis. Thus, 61.5% represents the proportion of participants who reported awareness among those with a valid response, not among all 4,700 participants.

Compared with participants aged < 30 years, those aged 30-50 years (adjusted Odds ratio (AOR) = 0.78; 95% CI: 0.66-0.92; p = 0.004) and >50 years (AOR = 0.54; 95% CI: 0.43-0.69; p < 0.001) had significantly lower odds of knowing dental implants. Female participants also demonstrated slightly lower odds of implant knowledge than male participants​​​​​ (AOR = 0.86; 95% CI: 0.75-0.98; p = 0.028). In contrast, higher educational attainment was significantly associated with greater knowledge, with diploma holders (AOR = 1.42), bachelor's degree holders (AOR = 2.08), master's degree holders (AOR = 2.81), and PhD holders (AOR = 3.74) showing progressively higher odds of implant awareness compared with participants having a high school education or below (all p < 0.001), as shown in Table 4.

**Table 4:** Binary logistic regression analysis for factors associated with knowledge of dental implants * indicates significance.

Variables	Category	Adjusted OR	95% CI	p-value
Age	<30 years	Reference	—	—
	30–50 years	0.78	0.66–0.92	0.004*
	>50 years	0.54	0.43–0.69	<0.001*
Gender	Male	Reference	—	—
	Female	0.86	0.75–0.98	0.028*
Educational level	High school or below	Reference	—	—
	Diploma	1.42	1.18–1.71	<0.001*
	Bachelor's degree	2.08	1.74–2.49	<0.001*
	Master's degree	2.81	2.16–3.66	<0.001*
	PhD	3.74	2.28–6.14	<0.001*

## Discussion

Dental implants have become an established treatment option for the replacement of missing teeth and rehabilitation of partially and completely edentulous patients. However, patients’ awareness, knowledge, expectations, and perceptions regarding implant treatment may influence their willingness to consider this treatment option [8]. Successful implant therapy depends not only on clinical expertise but also on patients' knowledge, expectations, and willingness to accept treatment. Previous studies have consistently demonstrated that implant-supported prostheses improve oral function, aesthetics, self-confidence, psychological well-being, and overall quality of life while preserving adjacent natural teeth [9,10].

The present study evaluated the knowledge, awareness, and attitudes of dental patients regarding dental implants as a treatment option for replacing missing teeth. Of the 5,000 eligible participants, 4,700 completed the questionnaire, yielding a response rate of 94%. Among the respondents, 3,358 (71.4%) had undergone tooth extraction, with dental caries being the leading cause of tooth loss (1,805; 53.8%). A substantial majority of these participants (3,102; 92.4%) expressed a willingness to replace their missing teeth, and 1,309 (42.2%) preferred dental implants as the treatment of choice, indicating a favorable attitude toward implant-supported rehabilitation. Awareness of dental implants was reported by 1,909 (61.5%) participants, while 1,193 (38.5%) were unaware of this treatment modality. Dentists emerged as the primary source of information, emphasizing their crucial role in patient education. Specifically, 1,341 of 4,700 participants (28.5%) reported an anticipated cost of ₹10,000-₹20,000. Financial considerations were therefore perceived as a major barrier to treatment acceptance.

In terms of maintenance, 1,589 (33.8%) participants believed that implants require greater oral hygiene care than natural teeth, while 1,298 (27.6%) considered the maintenance requirements to be similar. Overall, the findings suggest a moderate level of awareness and a positive attitude toward dental implants; however, misconceptions regarding cost and long-term maintenance highlight the need for comprehensive patient education and counseling. The findings of the present study are comparable with those reported by Al-Dwairi et al., who evaluated knowledge, awareness, and attitudes regarding dental implants among 5,000 dental patients using an 18-item questionnaire. They reported that approximately 75% of participants were aware of dental implants, with dentists, family members, friends, and mass media serving as the primary information sources. Their study further demonstrated a generally positive attitude toward implant therapy and emphasized that dentists play a central role in improving public awareness through patient education and counseling. Among the participants who responded to the specific awareness question (n = 3,102), the level of awareness in the present study was 61.5%, which was slightly lower than that reported by Al-Dwairi et al. Both studies indicate a generally favorable perception of dental implants and highlight the importance of professional guidance in improving patient knowledge [6].

Similarly, Al-Johany et al. evaluated awareness regarding dental implants among 379 dental patients in Riyadh, Saudi Arabia, and reported that 66.4% of participants were aware of implant therapy, which is comparable to the awareness observed in the present study. Friends and relatives constituted the most common source of information (31.5%), followed by dentists (28.3%). Furthermore, 82.4% of participants desired additional information regarding implants, with 85.2% preferring to receive such information directly from their dentists. Consistent with the present findings, treatment cost was identified as the principal barrier to implant acceptance (86.5%), followed by prolonged treatment duration and fear of surgery. These observations collectively suggest that although awareness of dental implants has improved, inadequate patient education and financial constraints continue to limit their widespread acceptance. Strengthening patient education programs and improving communication between clinicians and patients may substantially enhance the acceptance of implant-supported rehabilitation [11].

A major strength of the present study is its relatively large sample size of 4,700 participants, together with a high response rate. The study also evaluated several demographic factors and used multivariable logistic regression to examine the association of selected demographic characteristics with implant-related knowledge. These features provide a broader assessment of patients’ perceptions and awareness regarding dental implant treatment.

However, several limitations should be considered. First, this was a single-center, hospital-based cross-sectional study, and participants were recruited using consecutive non-probability sampling. Therefore, the findings may not be representative of the wider population and should be generalized with caution. Second, the questionnaire was modified from a previously published questionnaire, and the modifications may affect the comparability of the findings with studies using the original instrument. Third, several variables were based on self-reported responses, which may be influenced by recall or response bias. Finally, because of the cross-sectional design, associations identified between demographic characteristics and implant-related knowledge or perceptions should not be interpreted as causal relationships.

## Conclusions

The study demonstrated varying levels of awareness, knowledge, and attitudes toward dental implant treatment among partially edentulous dental patients. These perceptions were associated with demographic characteristics such as age, educational status, and financial status. Perceived treatment cost represented an important barrier to acceptance of implant therapy. The findings highlight the potential importance of clear patient education and individualized counseling to improve understanding of implant treatment, its benefits, limitations, and anticipated costs.
